# Role of long noncoding RNA taurine‐upregulated gene 1 in cancers

**DOI:** 10.1186/s10020-021-00312-4

**Published:** 2021-05-26

**Authors:** Miao Da, Jing Zhuang, Yani Zhou, Quan Qi, Shuwen Han

**Affiliations:** 1 Department of Oncology, Huzhou Central Hospital, Affiliated Central Hospital Huzhou University, No. 1558, Sanhuan North Road, Wuxing, Huzhou, Zhejiang People’s Republic of China; 2 Department of Nursing, Huzhou Third Municipal Hospital, 2088 East Tiaoxi Rd, Huzhou, Zhejiang People’s Republic of China; 3 Medical College of Nursing, Huzhou University, No. 759 Erhuan East Road, Huzhou, 313000 Zhejiang China; 4 Graduate School of Medicine Faculty, Zhejiang University, No. 866 Yuhangtang Road, Xihu, Hangzhou, 310058 Zhejiang People’s Republic of China; 5 Department of Oncology, Huzhou Central Hospital, Affiliated Central Hospital Huzhou University, No. 1558, Sanhuan North Road, Wuxing, Huzhou, 313000 Zhejiang China

**Keywords:** Long non-coding RNA (lncRNA), Taurine‐upregulated gene 1 (*TUG1*), Cancer, Competitive endogenous RNA (ceRNA), Biomarker

## Abstract

Long non-coding RNAs (lncRNAs) are a group of non-protein coding RNAs with a length of more than 200 bp. The lncRNA taurine up-regulated gene 1 (*TUG1*) is abnormally expressed in many human malignant cancers, where it acts as a competitive endogenous RNA (ceRNA), regulating gene expression by specifically sponging its corresponding microRNAs. In the present review, we summarised the current understanding of the role of lncRNA *TUG1* in cancer cell proliferation, metastasis, angiogenesis, chemotherapeutic drug resistance, radiosensitivity, cell regulation, and cell glycolysis, as well as highlighting its potential application as a clinical biomarker or therapeutic target for malignant cancer. This review provides the basis for new research directions for lncRNA *TUG1* in cancer prevention, diagnosis, and treatment.

## Introduction

Cancer is the second leading cause of death in the United States, with about 600,000 deaths each year (Farhad et al. 2020). Cancer epidemiology is evolving as a result of altered risk factor patterns, changes in disease classification, improved testing and treatment, and demographic changes, including aging, population growth, and migration (Farhad et al. 2020). Cancer remains a disease with high morbidity and mortality, and represents a serious threat to human health (Claudia et al. 2020). There are various signal pathways and molecules involved in the progression, invasion, and metastasis of cancer cells. However, the molecular mechanisms underlying cancer are complex, and have not yet been fully explained. Therefore, it remains important to explore the molecular mechanisms of cancer, particularly regarding novel diagnostic and therapeutic strategies.

With the rapid development of genomics technology, the important role of non-coding RNAs (ncRNAs) has been clarified in the processes of growth, development, and disease (Mitchell and Rinn 2012). Thousands of ncRNAs have been identified, including various small RNAs (such as microRNA (miRNA), small nuclear/nucleolar RNA, and piwi-interacting RNA), and a more uneven class of long non-coding RNAs (lncRNAs) (Taft et al. 2010). lncRNAs are a class of non-coding RNA molecules with a length of larger than 200 nt that have a mRNA-like structure, but do not encode a protein (Yihui et al. 2020). lncRNAs participate in embryogenesis, angiogenesis, and cancer progression by exerting epigenetic changes in many processes, including inactivation of X chromatin, regulation of the function of key metabolic genes, cell cycle control, and cell differentiation (Yao et al. 2019; Ding et al. 2020). Mounting evidence shows that lncRNAs are closely related to a variety of malignant cancers, and can play the role of oncogene or oncogene suppressor in different cancer types (Sisi et al. 2021; Miaomiao et al. 2021). For example, lncRNA MALAT1 promoted the progression of gastric cancer by inhibiting autophagy flux and inducing fibroblast activation (Zhenqiang et al. 2021). In renal cell carcinoma, HOTAIR and androgen receptors cooperatively increased GLI2 transcription to promote tumor angiogenesis and cancer stem cell property (Ji-Yu et al. 2021). Metastasis-associated protein 2 (MTA2) regulated by small nucleolar RNA host gene 5 (SNHG5) played an important role in the progression of oesophageal squamous cell carcinoma (Sisi et al. 2021).

Taurine upregulated gene 1 (*TUG1*), also known as TI-227H, LINC00080 and NCRNA00080, is located on the human chromosome 22 autosomal long arm 1 region 2 sub-band (22 q12.2), with a total length of about 7.1 kb (Tang et al. 2018). This molecule was first found in neonatal mouse retinal cells, where it promoted the development of the retina (Young et al. 2005). This lncRNA interacts with the polycomb repressive complex and plays a role in the epigenetic regulation of transcription. Recently, lncRNA *TUG1* research has mainly been focused on cancer, and lncRNA *TUG1* can regulate the development of cancers (Yihui et al. 2020). lncRNA *TUG1* is differentially expressed in cancers, and can affect the proliferation and apoptosis of cancer cells. The expression of lncRNA *TUG1* is closely related to the prognosis of cancer patients (Ding et al. 2020). lncRNA *TUG1* is thought to be involved in carcinogenesis and development mainly through competitive binding with miRNAs, regulation of cyclin-dependent kinase inhibitors, and effects on cancer proliferation and apoptosis (Young et al. 2005; Xiong et al. 2018) miRNAs are short RNAs that regulate a variety of physiological and biological processes in eukaryotic cells (Xia et al. 2020). miRNAs can bind to the 3′ untranslated region (3′-UTR) of the target gene mRNA, thus promoting mRNA cleavage or inhibiting mRNA translation (Li-Jun et al. 2017). Abnormal expression of lncRNA *TUG1* affected the proliferation, apoptosis, and invasion of a variety of cancers, including bladder urothelial carcinoma, osteosarcoma, non-small cell lung cancer, and oesophageal squamous cell carcinoma, suggesting that lncRNA *TUG1* may be used as a diagnostic marker or therapeutic target (Jun et al. 2016a; Qun et al. 2020).

**Fig. 1:**
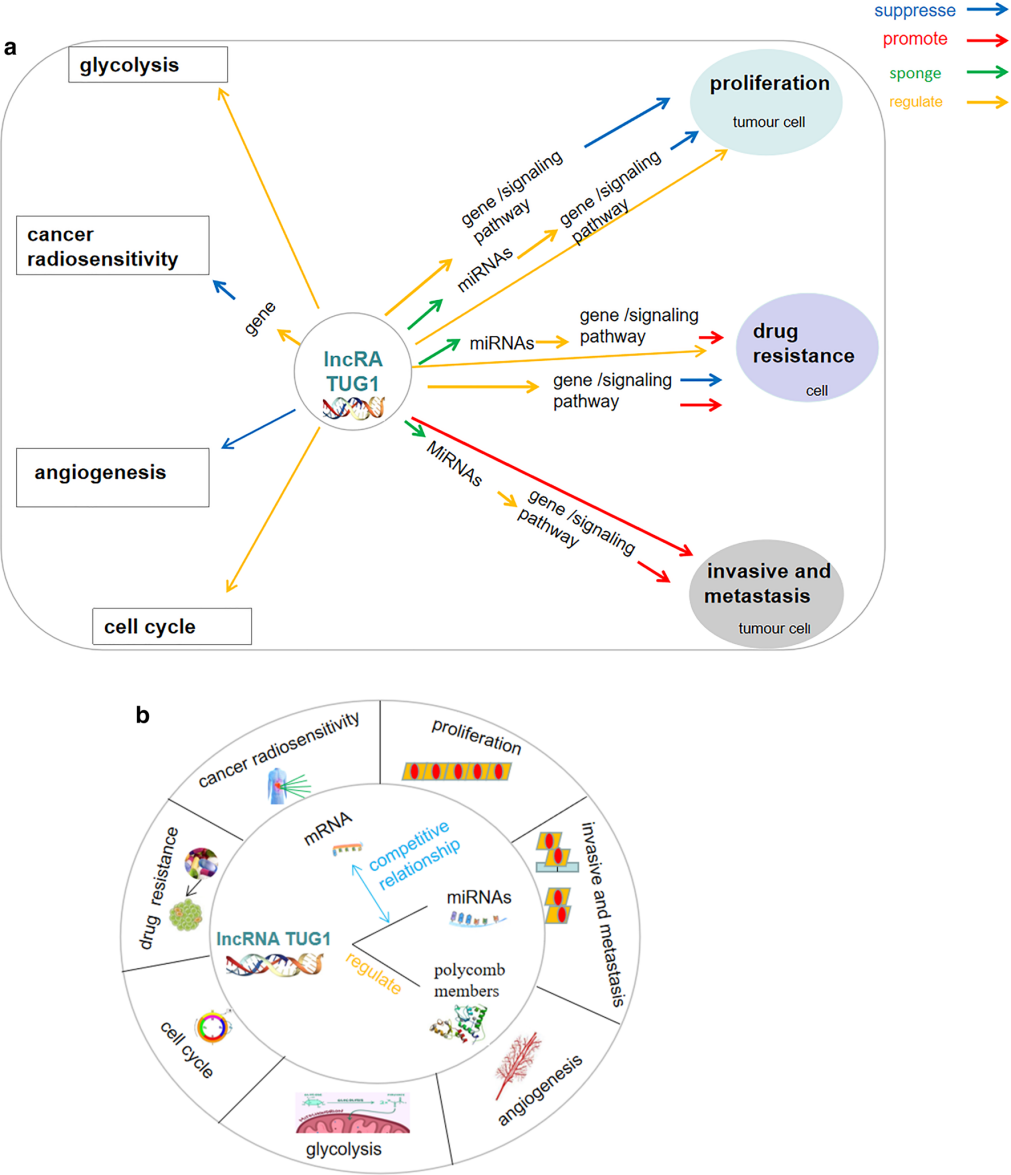
Diagram showing review outline. The present review summarises the roles of lncRNA TUG1 in the regulation of cancer cell proliferation, metastasis, angiogenesis, chemotherapeutic drug resistance, radiosensitivity, cell regulation, and cell glycolysis

**Fig. 2:**
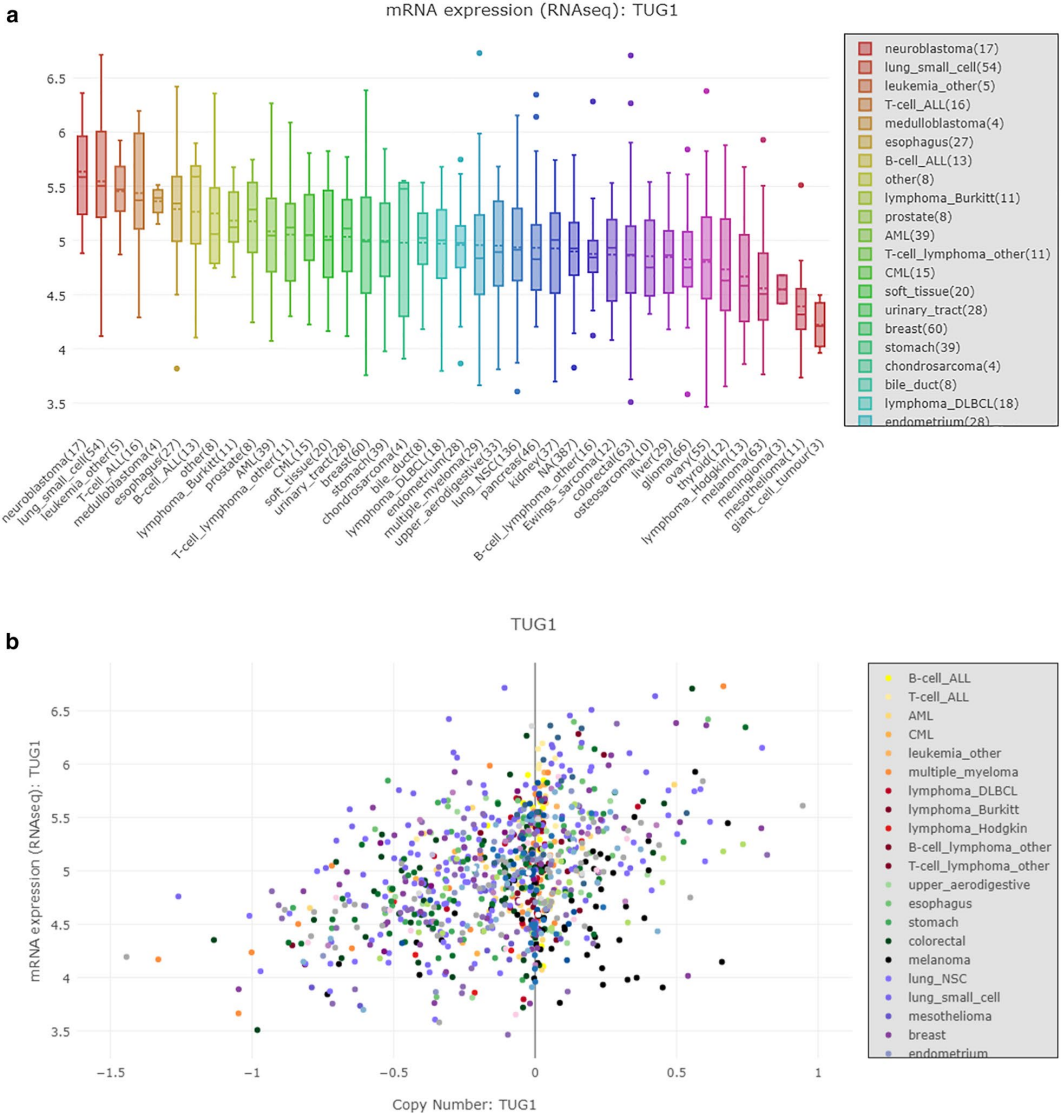
TUG1 expression in cancers. Figure 2 is publicly available data. **A** From the CCLE database, shows the expression of TUG1 in different cells. The box plot was sorted and colored basing on the average distribution of the expression of TUG1 in a lineage. The lineage consists of several cell lines from the same region or system of the body. The numbers next to lineage names indicate how many cells lines in the lineage. **B** is used to visually compared the TUG1 expression data with different data sets

In the present review, we evaluated the molecular mechanisms and clinical significance of lncRNA *TUG1* in different cancer types. We summarized the research progress of lncRNA *TUG1* in cancer cell proliferation, metastasis, angiogenesis, chemotherapeutic drug resistance, radiosensitivity, cell regulation, cell glycolysis, and its potential application as a clinical biomarker or therapeutic target for malignant cancer. A detailed summary of the review strategy is shown in Fig. 1. From Cancer Cell Line Encyclopedia (CCLE) database, Fig. 2 shows the expression of TUG1 in different cancers.

## Function of lncRNA *TUG1* in cancers

### lncRNA *TUG1* regulates cancer cell proliferation

lncRNA *TUG1* promoted cell proliferation in the digestive system. lncRNA *TUG1* promoted cell growth, proliferation, and invasion, and induced apoptosis of oral squamous cell carcinoma cells by targeting the Wnt/β-catenin signalling pathway (Shuang et al. 2017). lncRNA *TUG1* promoted the progression of nasopharyngeal carcinoma by enabling miR-384 to inhibit the epithelial-mesenchymal transformation (EMT) (Wei et al. 2019). lncRNA *TUG1* promoted the proliferation and invasion of oesophageal squamous cell carcinoma cells by regulating the expression of cell division cycle 42 (CDC42) through miR-498 (Zhifeng et al. 2020). lncRNA *TUG1*/miR-29c axis promoted the growth and migration of pancreatic cancer cells in vivo and in vitro (Yebin et al. 2018). lncRNA *TUG1*/miRNA-299-3p axis promoted the malignant progression of pancreatic cancer by inhibiting the Notch1 pathway (Ke and Lianfeng 2020).

lncRNA *TUG1* was also found in the urinary system. lncRNA *TUG1* promoted renal cell carcinoma formation via the miR-299-3p/vascular endothelial growth factor (VEGF) axis (Yunsheng et al. 2019). lncRNA *TUG1* expression by miR142-mediated zinc finger E-box binding homeobox 2 (ZEB2), through inactivating the Wnt/β-catenin pathway, promoted the proliferation of bladder cancer cells and induced apoptosis (Qian et al. 2017). Down-regulation of lncRNA *TUG1* inhibited the development and progression of prostate cancer by regulating the microRNA496/wnt/β-catenin pathway (Gang et al. 2020).

lncRNA *TUG1* had also been found in other systems. Direct lncRNA TUG1 promoted the proliferation and invasion of glioma cells, and promoted apoptosis (Zhao et al. 2018). lncRNA *TUG1* inhibited glioma cell proliferation (Jun et al. 2016a). In addition, lncRNA *TUG1* acted as miR-26a sponge to up-regulate the expression of phosphatase and tensin homolog (PTEN), and inhibited the development of glioma (Jun et al. 2016b). lncRNA *TUG1* was up-regulated in acute myeloid leukemia (AML) patients and cells, and lncRNA *TUG1* promoted the proliferation and glycolysis of AML cells by targeting miR-185 (Weide et al. 2020). lncRNA *TUG1* promoted the proliferation of AML cells and increased the rate of apoptosis (Jun et al. 2018a). lncRNA *TUG1* promoted the proliferation and invasion of osteosarcoma cells through sponging miR-153 (Wang Heping and Yanzhang 2018).

The expression of lncRNA *TUG1* was down-regulated in non-small cell lung cancer (Pei-Chin et al. 2016). lncRNA *TUG1* inhibited the proliferation of non-small cell lung cancer cells (Pei-Chin et al. 2016). The effect of p53-regulated lncRNA *TUG1* on the proliferation of non-small cell lung cancer cells was partly exerted through epigenetic regulation of homeobox B7 (HOXB7).(Zhang et al. 2014) lncRNA *TUG1* inhibited the proliferation non-small cell lung cancer (Zhang et al. 2014). lncRNA *TUG1* promoted the proliferation of MCF-7 breast cancer cells by inhibiting microRNA-9 (Xiao-Bo and Guo-Sheng 2016).

**Fig. 3:**
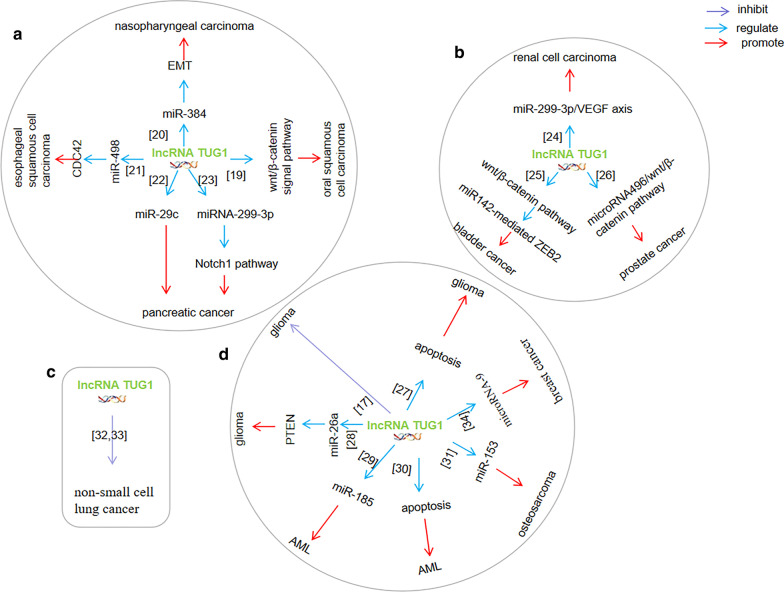
lncRNA TUG1 regulates cancer cells proliferation of a variety of cancers. A network map illustrating the regulation of proliferation by lncRNA TUG1 in a variety of cancers, including oral cancer, oesophageal cancer, and pancreatic cancer. The pie charts labelled **A**, **B**, **C** and **D** represent cancers of the digestive system, cancers of urinary system, non-small cell lung cancer and cancers of other systems, respectively

A summary of the regulation of cancer cell proliferation by lncRNA *TUG1* in a variety of cancers is presented in Fig. 3. lncRNA *TUG1* affected proliferation by regulating wnt/β-catenin signal pathway, miR-384, miR-498, miR-29c, miR-299-3p, or microRNA-9, in oral squamous cell carcinoma, nasopharyngeal carcinoma,esophageal squamous cell carcinoma, pancreatic cancer, breast cancer, respectively. lncRNA *TUG1* affected proliferation by miR-299-3p/VEGF pathway, ZEB2/miR-142/wnt/β-catenin pathway, microRNA496/wnt/β-catenin pathway in renal cell carcinoma, bladder cancer, prostate cancer, respectively. lncRNA *TUG1* affected proliferation by directly regulation, internal pathways mediated by caspase-3 and-9, anti-apoptotic pathways mediated by Bcl-2, or miR-26a sponging PTEN in glioma. lncRNA *TUG1* affected proliferation by targeting miR-185 or directly regulating in AML. lncRNA *TUG1* affected proliferation through miR-153 in osteosarcoma. However, the expression of lncRNA TUG1 was down-regulated in non-small cell lung cancer. lncRNA TUG1 inhibited proliferation of non-small cell lung cancer. Interestingly, the expression of lncRNA *TUG1* in lung cancer was very special. At present, the research objects of literature reports on lung cancer are mainly from China. It was possible that the biological behavior of different tumors affected the expression of lncRNA *TUG1*. This situation deserves further study.

### lncRNA *TUG1* regulates cancer invasion and metastasis

Up-regulation of lncRNA *TUG1* expression exerts a carcinogenic role by promoting the migration, and invasion of laryngeal cancer cells, and by inhibiting apoptosis (Zhang et al. 2018). lncRNA *TUG1* participated in the development of laryngeal carcinoma via inhibiting the activation of RhoA/rho associated coiled‐coil containing protein kinase (ROCK)/matrix metalloproteinase (MMPs) signalling pathway by miR-145-5p (Shenfa et al. 2019). Up-regulation of lncRNA TUG1 promoted the proliferation and migration of esophageal squamous cell carcinoma (Xu et al. 2015). lncRNA *TUG1* acts as ceRNA by adjusting the miR-1294/PLK1 axis (Mingzhu et al. 2020), targeting miR-498 to induce XBP1 expression (Jin et al. 2020), or regulating the Wnt/β-catenin pathway through the miR-148A-3p/mcl-1 axis (Yin et al. 2020) to promote growth and metastasis of oesophageal squamous cell carcinoma cells. lncRNA *TUG1* promoted the proliferation and invasion of gastric cancer cells by negatively modulating miRNA-145-5p (Kewei et al. 2017). Overexpression of lncRNA *TUG1* contributed to the proliferation and migration of colon cancer cells (Hui-Yuan et al. 2016). lncRNA *TUG1* also regulates invasion and metastasis through other mechanisms, such as via miR-145-5p/TRPC6 (Xiaoqiang et al. 2021), the miR-26a-5p/matrix metalloproteinases-14 (MMP-14)/p38 mitogen-activated protein kinase (p38MAPK)/HSP27 (heat shock protein 27) axis (Lei et al. 2019) the *TUG1*/Twist1/EMT signalling pathway ,(Shen et al. 2020), or by affecting the EMT (Liang et al. 2016) to promote proliferation and metastasis in colorectal cancer cells. The lncRNA *TUG1*/enhancer of zeste homolog 2 (EZH2) axis promoted the proliferation, migration, and EMT phenotype of pancreatic cancer cells by sponging miR-382 (Liang et al. 2017).

To sum up, lncRNA *TUG1* promoting invasion and metastasis by some signal pathways, including RhoA/ROCK/MMPs axis, miR-1294/PLK1 axis, miR-498/XBP1 axis, miR-148A-3p/mcl-1/Wnt/β-catenin axis, miR-145-5p/TRPC6 axis, miR-26a-5p/MMP-14/p38MAPK/HSP27 aix, or Twist1/EMT axis, and some molecule, including miR-145-5p, EMT, EZH2, or miR-382 in cancers.

lncRNA *TUG1* promoted the development of osteosarcoma through runt-related transcription factor 2 (RUNX2) (Kunkun and Yan 2019). lncRNA *TUG1* regulated the proliferation and invasion of osteosarcoma through a variety of different miRNA, such as hypoxia inducible factor-1α mediated by miR-143p (Yu Xiao and Lei 2019), sponging miR-9-5p and regulation of POU2F1 expression (Chu-Hai et al. 2016), miR-212-3p/forkhead box A1 (FOXA1) axis (Chuhai et al. 2018), miR-140-5p/PFN2 axis (Zhao et al. 2019), inhibition of microRNA-212-3p expression (Heng et al. 2018), miR-335-5p on Rho-associated coiled-coil containing protein kinase 1 (ROCK1) expression and ROCK1-mediated expression (Yong et al. 2017), or sponging of miR-132-3p and up-regulation of sex determining region Y-box 4 (SOX4) expression (Gang et al. 2018). There was also a positive and negative feedback regulation mode for lncRNA *TUG1* in osteosarcoma. Forkhead Box M1 (FOXM1) up-regulated the expression of lncRNA *TUG1* in osteosarcoma cells (Yang et al. 2018a). lncRNA *TUG1* promoted the proliferation, migration, and invasion of osteosarcoma through competitively sponging of miR-219a-5p, resulting in the up-regulation of phosphatidylinositol-4, 5-bisphosphate 3-kinase catalytic subunit alpha (PIK3CA) and the activation of the protein kinase B (AKT) signal pathway (Yang et al. 2018a). In addition, the activation of the AKT pathway promoted the expression of lncRNA *TUG1* by up-regulating the expression of FOXM1, forming a positive feedback loop in osteosarcoma (Yang et al. 2018a).

To sum up, lncRNA *TUG1* promoted invasion and metastasis by some molecule, including miR-143p, miR-9-5p, miR-212-3p, miR-219a-5p, miR-140-5p, miR-212-3p, miR-335-5p, miR-132-3p, SOX4, RUNX2, PFN2, ROCK1,FOXA1, or POU2F1 in osteosarcoma.

lncRNA *TUG1* promoted the growth and metastasis of cholangiocarcinoma cells by inhibiting miR-29a (Yuan et al. 2020). lncRNA *TUG1* sponging of miR-145 promoted intrahepatic cholangiocarcinoma progression, and regulated glutamine metabolism through the signal transducer and activator of transcription 3 (STAT3)/GDH axis (Bing et al. 2017). lncRNA *TUG1* promoted hepatoma cell proliferation, migration and invasion, inhibited apoptosis, and up-regulated AURKA expression in hepatocellular carcinoma (Peng et al. 2018a). lncRNA *TUG1* interacted with a variety of miRNAs to promote hepatoma cell migration, and invasion, such as miR-144 through activation of the Janus kinase 2 (JAK2)/STAT3 pathways (Jun et al. 2018b), the miR-216b-5p/DLX2 axis (Qun et al. 2020), downregulation of miR-142-3p (Chuan et al. 2018), or the miR-29c-3p/ collagen type 1 alpha 1 (COL1A1) axis (Wei et al. 2020).

lncRNA *TUG1* knockout promoted cell growth by promoting cell cycle progression and regulating the expression of cyclinD1 and CDK4 (Fan et al. 2017). lncRNA *TUG1* suppressed miR-196A (Yang et al. 2018b), regulated the MIR-31-5p/flotillin 1 (FLOT1) axis (Dong et al. 2020), or regulated yes‐associated protein (YAP) (Shan et al. 2018) to promote the proliferation and migration of renal cell carcinoma. The upregulation of lncRNA *TUG1* promoted the proliferation, migration, and invasion of bladder cancer cells by inhibiting miR-29c (Peng et al. 2018b). lncRNA *TUG1*, as the ceRNA of miR-26a, promoted the progression of prostate cancer (Bin et al. 2018). lncRNA *TUG1* accelerated the progression of prostate cancer by regulating the MIR-128-3p/YES1 axis (Hao et al. 2020).

Up-regulation of lncRNA *TUG1* promoted the proliferation and migration of cervical cancer cells (Yingying et al. 2017). lncRNA *TUG1* predicted a poor prognosis in epithelial ovarian cancer, promoted cell proliferation, and inhibited apoptosis (Tong-Huai et al. 2018). lncRNA *TUG1* also regulated aurora kinase A (AURKA) (Tonghuai et al. 2018), or sponged miR-1299 by up-regulating notch receptor 3(NOTCH3) (Yuqing et al. 2020) to promote the proliferation and invasion of epithelial ovarian cancer cells. lncRNA *TUG1* promoted the progression of ovarian cancer by targeting the mir-29b-3p/MDM2 axis (Xiaoqiu et al. 2020).

In AML patients, lncRNA *TUG1* was associated with disease progression and poor prognosis, induced AML cell proliferation, and inhibited apoptosis by targeting AURKA (Xinfeng et al. 2018). lncRNA *TUG1* also promoted the proliferation, migration, and invasion of AML cells by regulating miR-370-3p/MAPK1/ERK (Gang et al. 2019).

**Fig. 4:**
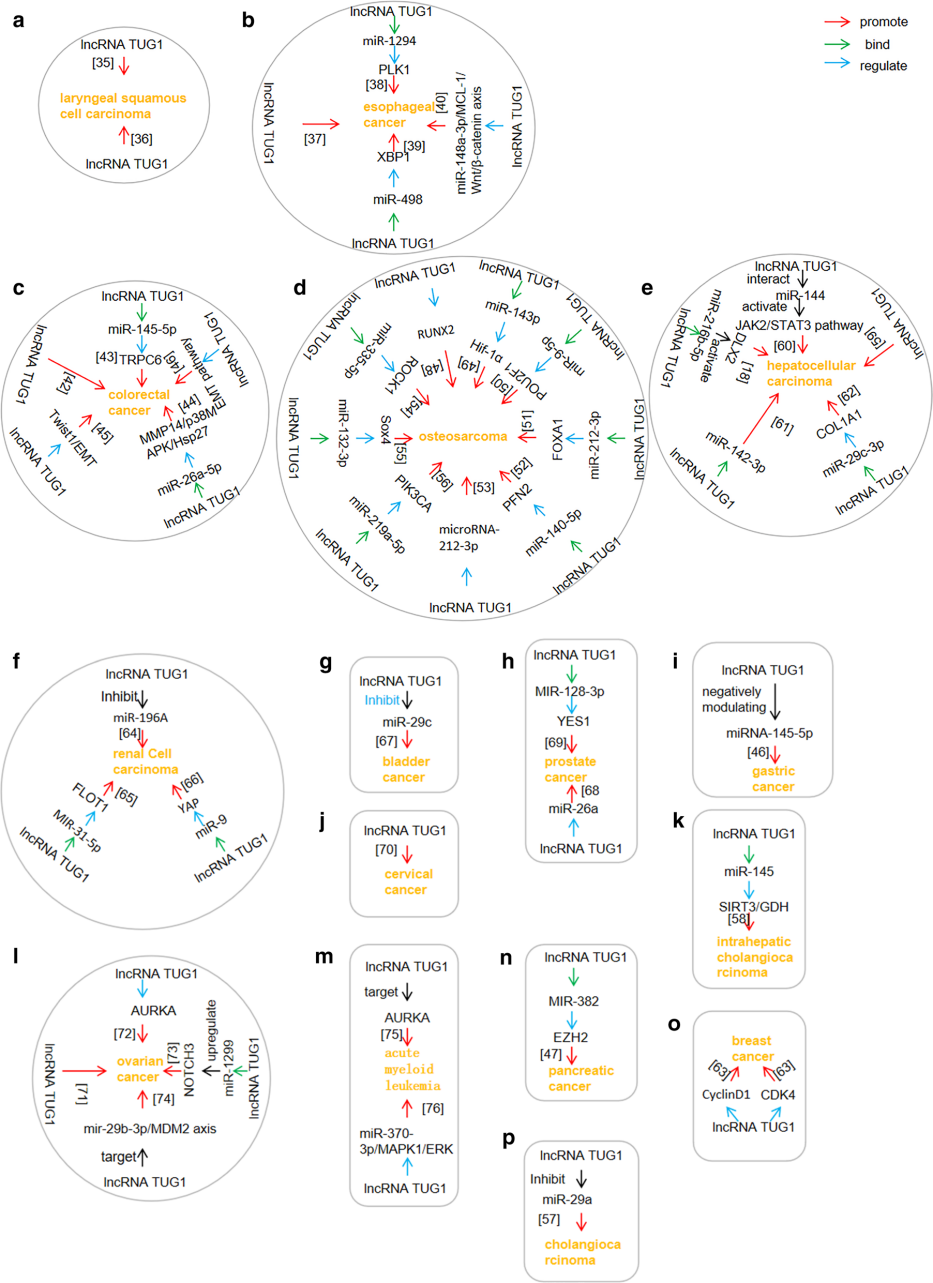
lncRNA TUG1 regulates cancer invasion and metastasis in many different cancer types. A diagram showing the regulation of cancer invasion and metastasis by lncRNA TUG1 in different cancer types. The pie charts labelled **A–Q** represent laryngeal squamous cell carcinoma, oesophageal cancer, non-small cell lung cancer, colorectal cancer, osteosarcoma, hepatocellular carcinoma, renal cell carcinoma, bladder cancer, prostate cancer, gastric cancer, cervical cancer, intrahepatic cholangiocarcinoma, ovarian cancer, acute myeloid leukemia, pancreatic cancer, breast cancer, and cholangiocarcinoma, respectively

Figure 4 summarises the regulation of invasion and metastasis by lncRNA *TUG1* in a variety of cancers.

### lncRNA *TUG1* regulates cell cycle

The progression of the cell cycle is controlled by the induction of cyclins and the activation of homologous cyclin-dependent kinases (Xiao et al. 2020). lncRNA *TUG1* acts on amplified in breast cancer 1 (AIB1) to regulate the cell cycle in ovarian cancer (Li et al. 2017a). The expression of G1 phase related proteins was significantly changed (Li et al. 2017a). In tongue squamous cell carcinoma, down-regulation of lncRNA *TUG1* inhibited cell proliferation, and silencing of lncRNA *TUG1* regulated the progression of the cell cycle (Li et al. 2017b). *TUG1* knockout blocked cell cycle, accelerated apoptosis and inhibitted the proliferation of pancreatic cancer cells (Hui Bingqing and Yetao 2019). Knocking out the *TUG1* reduced that enhancer of zeste homolog 2 (EZH2) binded to the promoter regions of Rho family GTPase 3 (RND3) and metallothionein 2A (MT2A) (Hui Bingqing and Yetao 2019).

### lncRNA *TUG1* regulates cellular glycolysis

Glycolysis is an oxygen-independent metabolic pathway. In this process, glucose is converted to pyruvate, which then produces lactic acid (Ozcan Selahattin et al. 2020). Glycolysis reflects a change in the energy metabolism of cancer cells. In the presence of oxygen, malignant cells have higher glycolysis rates than normal cells, which is known as the "Warburg Effect" (Ozcan Selahattin et al. 2020; Bensinger and Christofk 2012). lncRNA *TUG1* knockout inhibited glucose consumption, lactic acid production, and reduced the cell viability of osteosarcoma cells. Overexpression of lncRNA *TUG1* increased cell viability, while 2-deoxy-D-glucose (2-DG) could attenuate this increase. The abnormal expression of lncRNA *TUG1* significantly affected the expression of hexokinase-2 (HK2), which might be an important molecule through which lncRNA *TUG1* affects glycolysis (Xiufu et al. 2018). HK2 gene knockout weakened the effect of lncRNA *TUG1* overexpression on glycolysis in osteosarcoma cells (Xiufu et al. 2018). lncRNA *TUG1* was up-regulated in AML patients and cells, and its knockout inhibited glycolysis in AML cells by targeting miR-185 (Weide et al. 2020).

### lncRNA *TUG1* regulates cancer angiogenesis

Angiogenesis is one of the prerequisites for active cancer progression. Under the regulation of hormones, including VEGF, angiogenesis plays a key role in the pathogenesis of ovarian cancer (Protopsaltis Nicholas et al. 2019). lncRNA *TUG1* induced the expression of VEGF, cancer growth factor-α, and angiopoietin-1 in vascular endothelial cells through leucine-rich α-2-glycoprotein-1 (LRG1) (Mingjun et al. 2019). Knockout of lncRNA *TUG1* inhibited angiogenesis in ovarian cancer by regulating LRG1 (Mingjun et al. 2019).

### lncRNA *TUG1* regulates resistance and sensitivity to chemotherapeutic drugs

Chemotherapy resistance remains the limiting factor in the treatment of cancer (Daniela and Rosario 2020). lncRNA *TUG1* inhibited the expression of PDCD4 through epigenetic pathways (Caihui et al. 2018), or by up-regulating nuclear factor (erythroid-derived 2)-like 2 (Nrf2) (Zhenghua et al. 2019), to make confer cisplatin resistance in oesophageal squamous cell carcinoma. lncRNA *TUG1* regulated CCND2, through EZH2-related miR-194-5p silencing, to promote the growth of bladder cancer cells and confer cisplatin resistance (Gan et al. 2019). Low expression of lncRNA *TUG1* enhanced the sensitivity of cervical cancer to cisplatin by activating the MAPK pathway (Xuemin et al. 2019). Down-regulation of lncRNA *TUG1* inhibited cisplatin resistance in drug-resistant tongue squamous cell carcinoma cells, by mediating miR-133b and cysteine-X-cysteine chemokine receptor 4 (CXCR4) (Ke et al. 2020). lncRNA *TUG1* knockout can induce apoptosis by inhibiting MET/Akt signalling, thus reducing the resistance of osteosarcoma cells to cisplatin (Zhou Qiang and Yuan 2020).

Up-regulation of lncRNA *TUG1* expression in bladder urothelial carcinoma inducing by transcription factor Nrf2 promoted cancer progression and adriamycin resistance (Zhulei et al. 2019). Polydatin inhibition of Akt signalling, mediated by lncRNA *TUG1*, suppressed the proliferation of doxorubicin-resistant osteosarcoma and promoted its apoptosis (Tongzhou et al. 2019). lncRNA *TUG1* induced autophagy of ovarian cancer cells by targeting miR-29b-3p, which lead to drug resistance to paclitaxel (Lize et al. 2020).

lncRNA *TUG1* enhanced adriamycin resistance in AML by inhibiting the expression of miR-34a through EZH2 epigenetically (Li et al. 2019). lncRNA *TUG1* reduced the sensitivity of AML cells to cytarabine by regulating the miR-655-3p/cyclin D1 (CCND1) axis (Zhang et al. 2020).

**Fig. 5:**
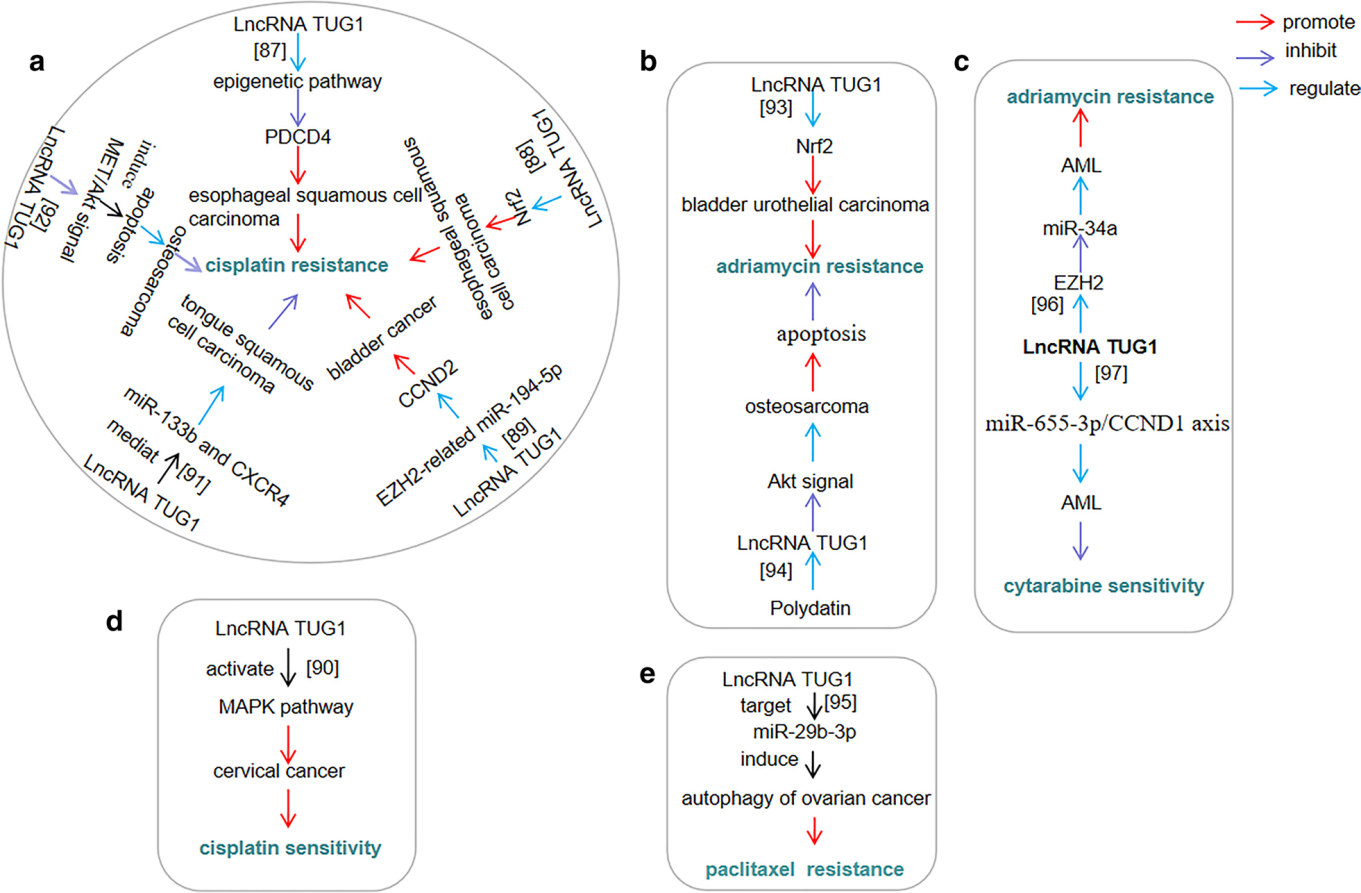
lncRNA TUG1 regulates resistance and sensitivity to chemotherapeutic drugs. A diagram showing the regulation of resistance and sensitivity to chemotherapeutic drugs by lncRNA TUG1 in different cancer types. The pie charts labelled **A**, **B**, **C**, **D**, and **E** represent cisplatin resistance, adriamycin resistance, cytarabine sensitivity, cisplatin sensitivity, and paclitaxel resistance, respectively

Figure 5 shows a summary of the regulation of resistance and sensitivity to chemotherapeutic drugs in different cancers by lncRNA *TUG1*.

### lncRNA *TUG1* regulates radiosensitivity

Radiotherapy is an effective strategy to prevent cancer metastasis. However, radiation resistance in cancer can limit the efficiency of radiotherapy (He Pan and Yong-Qiang 2020). The expression of lncRNA *TUG1* was up-regulated in bladder cancer tissues and cell lines. The down-regulation of lncRNA *TUG1* enhanced the radiosensitivity of bladder cancer cells by inhibiting the expression of high mobility group box-1 protein (HMGB1) (Jiang Huijuan and Xigang 2017). Knockout of lncRNA *TUG1* enhanced the radiosensitivity of prostate cancer through the lncRNA *TUG1*/miR-139-5p/structural maintenance of chromosomes protein 1A (SMC1A) axis (Dianhui et al. 2020). lncRNA *TUG1* up-regulated the expression of MET through sponging miR-144-3p, which activated the AKT signalling pathway and enhanced the radiosensitivity of oesophageal carcinoma (Pan et al. 2020).

## Clinical studies of lncRNA *TUG1* in cancers

Current clinical studies of lncRNA *TUG1* are shown in Table 1. The high expression of lncRNA *TUG1* was associated with chemotherapy resistance and poor prognosis in oesophageal squamous cell carcinoma (Lin et al. 2016). Conversely, the expression of lncRNA *TUG1* in viral hepatitis C and viral hepatitis C-associated hepatocellular carcinoma decreased (Mai et al. 2020). lncRNA *TUG1* was used as a non-invasive, cost-effective, and complementary biomarker in viral hepatitis C and viral hepatitis C-associated hepatocellular carcinoma (Mai et al. 2020). The level of alpha-fetoprotein (AFP) in patients with non-hepatitis B/non-C hepatocellular carcinoma (NBNC-HCC) was positively correlated with that of lncRNA *TUG1*, and the prognosis was poor (Lin et al. 2020). lncRNA *TUG1* may be an effective prognostic marker of NBNC-HCC (Lin et al. 2020). The expression of lncRNA *TUG1* in male lung cancer tissues was lower than that in the corresponding paracancerous tissue (Farbod and Mohammad 2019). The expression of lncRNA *TUG1* was down-regulated and used as a diagnostic marker in bladder cancer tissues (Feraydoon et al. 2020). lncRNA *TUG1* can be used to predict the resistance of ovarian cancer patients to cisplatin (Nashwa et al. 2020). Elevated lncRNA *TUG1* levels was a potential biomarker for the diagnosis of multiple myeloma (Qingqing et al. 2019). The expression of lncRNA *TUG1* in osteosarcoma was significantly higher than that in adjacent normal bone tissue (Qunli and Qi 2018). The up-regulation of lncRNA *TUG1* expression was significantly correlated with larger tumour size and late stages of lymph node metastasis in patients with osteosarcoma (Qunli and Qi 2018). However, low expression levels of lncRNA *TUG1* was an independent indicator of poor prognosis in patients with osteosarcoma (Qunli and Qi 2018). lncRNA *TUG1* was highly expressed in refractory or relapsed acute myeloid leukaemia (R/R AML), and may be a potential biomarker of poor prognosis in patients with R/R AML treated with granulocyte colony-stimulating factor (G-CSF) (CLAG) or fludarabine combined with cytarabine and G-CSF (FLAG) chemotherapy (Luo Wenfeng and Huilan 2018).

**Table 1:** Expression and clinical significance of lncRNA TUG1 in human cancer

No	Year	Authors	Cancer	Findings	Methods	Marker	References
1.1	2020	Mai Mohyeldeen, et al.	Hepatocellular carcinoma	Expression of lncRNA TUG1 was down-regulated in viral hepatitis C and viral hepatitis C-associated hepatocellular carcinoma and was closely associated with deregulated liver function and elevated AFP levels	Liver function tests, the antioxidant status, serum AFP and TUG1	Diagnosis	(Mai et al. 2020)
1.2	2020	Yang-Hsiang Lin, et al.	Hepatocellular carcinoma	AFP mRNA levels showed strong positive correlations with lncRNA TUG1 and unfavorable prognosis in patients with non-hepatitis B/non-hepatitis C hepatocellular carcinoma	Quantitative real-time polymerase chain reaction (qRT-PCR)	Progression	(Lin et al. 2020)
1.3	2020	El-Khazragy Nashwa, et al.	Ovarian cancer	The expression of lncRNA TUG1 was down-regulated in cisplatin-resistant tissues	qRT-PCR	Cisplatin resistance	(Nashwa et al. 2020)
1.4	2020	Abdolmaleki Feraydoon, et al.	Bladder cancer	Expression of lncRNA TUG1 was down-regulated in bladder cancer tissues	qRT-PCR	Diagnosis	(Feraydoon et al. 2020)
1.5	2019	Qingqing Yin, et al.	Multiple myeloma	Expression of lncRNA TUG1 was high in multiple myeloma	qRT-PCR	Diagnosis	(Qingqing et al. 2019)
1.6	2019	Farbod Esfandi, et al.	Non-small cell lung cancer	Expression of lncRNA TUG1 was down-regulated in tumoral tissues obtained from male subjects compared with the corresponding adjacent non-cancerous tissues	qRT-PCR, In silico analyses	Diagnosis	(Farbod and Mohammad 2019)
1.7	2018	Qi Chen, et al.	Osteosarcoma	Expression of lncRNA TUG1 was high in tumor tissues and it was association with carcinogenesis and progression in osteosarcoma	qRT-PCR	Progression	(Qunli and Qi 2018)
1.8	2018	Wenfeng Luo, et al.	R/R AML	Expression of lncRNA TUG1 was high in R/R AML patients. It might serve as a potential biomarker for poor prognosis in R/R AML patients treated with CLAG or FLAG based chemotherapy	qRT-PCR*,* Statistical analysis	Progression	(Luo Wenfeng and Huilan 2018)
1.9	2016	Lin Jiang, et al.	Esophageal squamous cell carcinoma	Expression of lncRNA TUG1 was high in esophageal squamous cell carcinoma tissues. It was correlated with chemotherapy resistance and might predict a poor prognostic outcome of esophageal squamous cell carcinoma	qRT-PCR	Progression	(Lin et al. 2016)

Sum up, lncRNA *TUG1* have been used as a biomarker in esophageal squamous cell carcinoma, viral hepatitis C and viral hepatitis C-associated hepatocellular carcinoma, NBNC-HCC, lung cancer, bladder cancer, ovarian cancer, osteosarcoma and R/R AML.

## Discussion

At present, the literature reviews on lncRNA *TUG1* are mainly based on the classification of different cancers. In the present review, we have comprehensively analysed and classified the literature related to lncRNA *TUG1* into its molecular mechanisms and clinical research categories. The molecular mechanisms were mainly summarized based on cancer cell proliferation, metastasis, angiogenesis, chemotherapeutic drug resistance, radiosensitivity, cell regulation, and cell glycolysis. At present, there are relatively fewer clinical studies on lncRNA *TUG1*, but existing studies suggest that lncRNA *TUG1* may be an effective diagnostic or prognostic cancer biomarker.

**Fig. 6:**
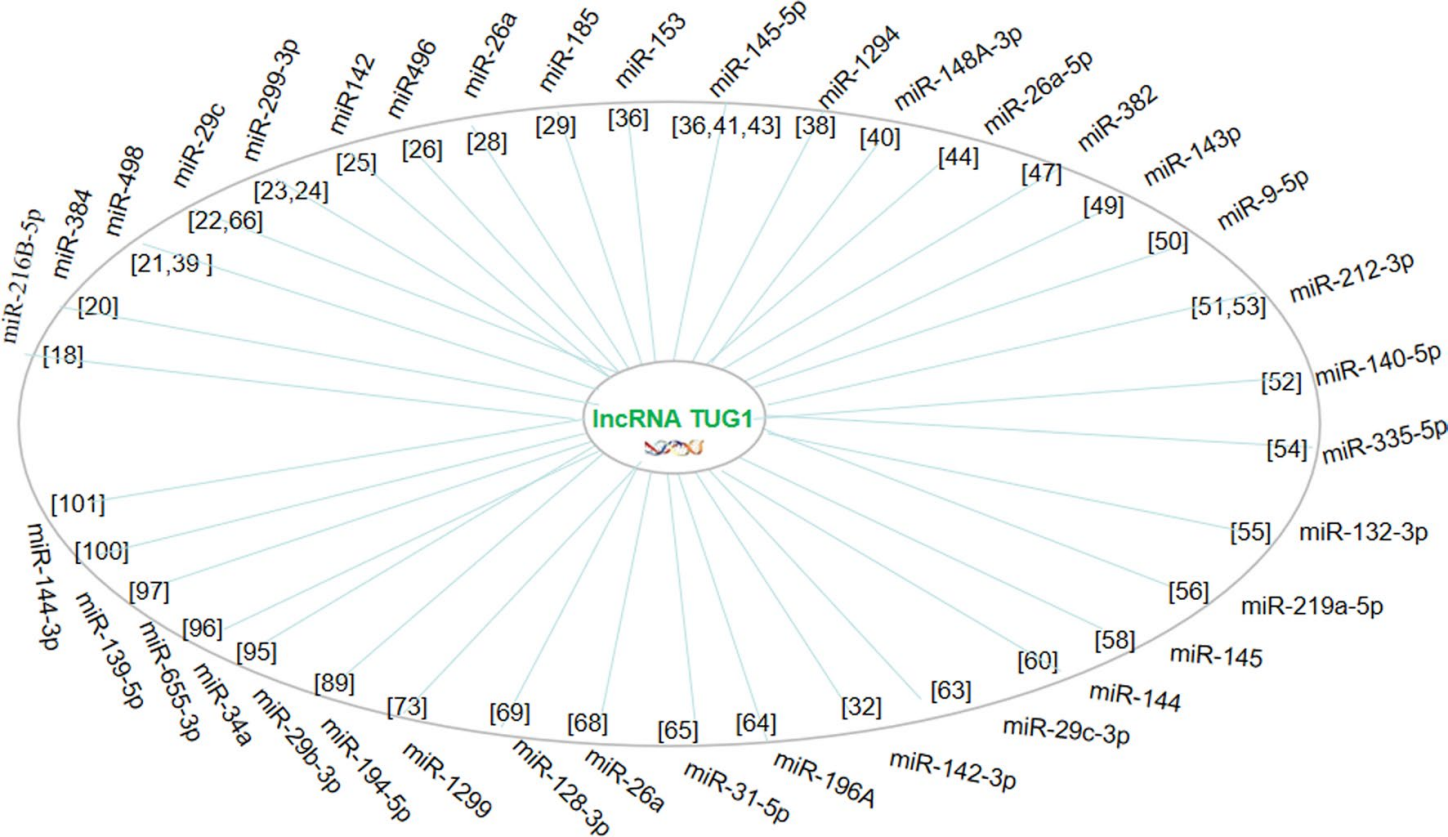
lncRNA TUG1 acts as ceRNA to compete with miRNAs. A diagram summarising the activity of lncRNA TUG1 as a ceRNA to compete with miRNAs in different cancer types

lncRNAs can act as ceRNAs to inhibit the function of miRNAs by preventing the interaction between miRNAs and their target mRNAs, thus affecting the translation of protein-coding genes (Zhang et al. 2020). Fig. 6 shows the miRNAs that are targeted by lncRNA *TUG1* as a ceRNA to regulate cancer growth, metastasis, angiogenesis, and chemotherapeutic drug resistance.

lncRNA *TUG1* promoted the proliferation of cancers, such as oral squamous cell cancer, bladder cancer, and prostate cancer. However, it had an inhibitory effect on the proliferation of non-small cell lung cancer. *TUG1* played different roles in different cancers. It might be related to cancer biological behavior and other related regulatory factors. lncRNA *TUG1* promoted the migration, invasion and metastasis of laryngeal cancer, esophageal squamous cell cancer and gastric cancer. lncRNA *TUG1* induced cycle arrest in pancreatic cancer cells and promoted apoptosis. The glycolysis process of acute myelogenous leukemia was affected by lncRNA *TUG1* to promote cancer growth. It was also stimulative for angiogenesis of ovarian cancer. Therefore, lncRNA *TUG1* played a role in cancer cell proliferation, migration, invasion, cell cycle, angiogenesis and glycolysis. In esophageal squamous cell cancer and bladder cancer, lncRNA *TUG1* was involved in the occurrence of cisplatin resistance, and the development of azithromycin resistance in urothelial cancer of the bladder and acute myelogenous leukemia. Therefore, lncRNA *TUG1* was involved in the process of chemotherapeutic drug resistance in different cancers. Although lncRNA *TUG1* could reduce the radiosensitivity of bladder cancer, it could also enhance the radiosensitivity of esophageal cancer. This paradox suggested that lncRNA *TUG1* had different effects on the radiosensitivity of different cancers. It might be related to cancer heterogeneity, cancer microenvironment or immune system.

lncRNA *TUG1* can regulate a variety of signal pathways, especially the Wnt/β-catenin pathway, during the development of cancer. As a highly conserved and tightly regulated signal pathway, Wnt/β-catenin plays an important role in the regulation of embryonic development, cell proliferation, and differentiation (Zhiqiang et al. 2020). If any of the key proteins in this signalling pathway are mutated, resulting in abnormal signal activation, it may induce the development of cancer (Shuang et al. 2017). For example, activating the Wnt/β-catenin signalling pathway regulated the invasion and proliferation of oesophageal squamous cell carcinoma, cervical cancer, bladder cancer, and colorectal cancer, and induced their epithelial cell transformation (Fu-Bing et al. 2020). In accordance, lncRNA *TUG1* can regulate the growth, proliferation, and invasion of oral squamous cell carcinoma, bladder cancer, and prostate cancer through the Wnt/β-catenin signalling pathway. The main components of Wnt signal pathway include secretory protein Wnt family, transmembrane receptor Frizzled family, Axin, β-Catenin, and transcription factor TCF/LEF family, etc. However, it is not clear which components are involved in the regulation of wnt signal pathway by lncRNA *TUG1*, which still need to be further explored.

In addition to Wnt signalling, lncRNA *TUG1* can also regulate the development of cancers through other pathways. lncRNA *TUG1* promoted the proliferation of oesophageal squamous cell carcinoma cells by regulating the miR-1294/PLK1 axis, either by inducing XBP1 expression via miR-498, regulating the Wnt/β-catenin pathway via the miR-148A-3p/mcl-1 axis, or by direct up-regulation. lncRNA *TUG1* promoted the proliferation and migration of colorectal cancer cells through the miR-145-5p/TRPC6 pathway, EMT pathway, miR-26a-5p/MMP14/p38MAPK/Hsp27 axis, Twist1/EMT signal pathway, or by overexpression. lncRNA TUG1 promoted the proliferation, migration, and invasion of hepatoma cells by activating the JAK2/STAT3 pathway, upregulating the expression of AURKA, interacting with miR-216B-5p and inhibiting apoptosis by activating DLX2, or by down-regulating miR-142-3p to regulate the miR-29c-3p/COL1A1 axis. lncRNA *TUG1* regulated downstream genes, including miR-143p, miR-9-5p, miR-212-3p, miR-140-5p, microRNA-212-3p, miR-335-5p and miR-219a-5p, to participate in the proliferation and invasion of osteosarcoma cells. There are multiple regulatory pathways in each kind of tumor, but further molecular experimental studies are still needed to clarify the interactions and relationships between various pathways.

### Future research direction

The functions of lncRNAs are diverse and complex. lncRNAs can regulate gene expression at different stages, steps, and levels, including epigenetically, transcriptionally, post-transcriptionally, and via miRNA. There are four main modes of action of lncRNA, denoted signal, decoy, guide, and scaffold. After decoy lncRNAs are transcribed, they bind to RNAs (ceRNA)/protein (transcription factor/transcriptional regulator), thus blocking the action of the RNA molecule. Currently, it has been established that lncRNA *TUG1* acts as a decoy lncRNA to exert its role in cancers. However, future studies still need to explore other possible mechanisms of action for lncRNA *TUG1*. At present, the molecular and clinical studies on lncRNA *TUG1* are in their infancy. Although studies suggest that lncRNA *TUG1* mainly regulates miRNAs downstream, there remains a lack of specific types of molecular experiments to verify these. Further molecular experiments should be carried out to expand the role of lncRNA *TUG1* in different cancer types, and to explore its role in body fluids. A more complete overview of the role of lncRNA *TUG1* in regulating miRNA in different cancers could provide guidance for the diagnosis and treatment of cancers in future. In addition, established machine learning models based on a large number of sequencing data can predict the molecular regulatory network with regard to lncRNA *TUG1* in cancers. This can provide a new direction for the future.

## Conclusion

It is a long and complicated process to explore the relation between lncRNA *TUG1* and cancer. In the present review, we summarized the available literature to show that lncRNA *TUG1* can regulate cancer cell proliferation, metastasis, angiogenesis, chemotherapeutic drug resistance, radiosensitivity, cell regulation, and cell glycolysis by regulating multiple molecular signalling pathways. This lncRNA could be used as a potential molecular target for cancer diagnosis and treatment in the future. The evaluation of the expression levels and functions of lncRNA *TUG1* in cancer requires further research to provide a reference for accurate targeted therapy.

## Data Availability

Not applicable.

## Abbreviations

AKT: Activation of protein kinase B
AURKA: Aurora kinase A
AML: Acute myeloid leukemia
AIB1: Amplified in breast cancer 1
AFP: Alpha-fetoprotein
ceRNA: Competitive endogenous RNA
CDC42: Cell division cycle 42
COL1A1: Collagen type 1 alpha 1
CELF1: CUGBP and Elav-like family member 1
CXCR4: Cysteine-X-cysteine chemokine receptor 4
CCLE: Cancer Cell Line Encyclopedia
G-CSF: Granulocyte colony-stimulating factor
EMT: Epithelial-mesenchymal transformation
EZH2: Enhancer of zeste homolog 2
FLAG: Fludarabine combined with cytarabine and G-CSF
FOXA1: Forkhead box A1
FOXM1: Forkhead Box M1
FLOT1: Flflotillin 1
HSP27: Heat shock protein 27
HMGB1: High mobility group box 1 protein
HK2: Hexokinase-2
HOXB7: Homeobox B7
HOTAIR: Hox transcript antisense intergenic RNA
JAK2: Janus kinase 2
LRG1: Leucine-rich α-2-glycoprotein-1
lncRNAs: Long non-coding RNAs
MT2A: Metallothionein 2A
MiRNA: MicroRNA
MMPs: Matrix metalloproteinases
MMP-14: Matrix metalloproteinases-14
MAPK1: Mitogen-activated protein kinase 1
MTA2: Metastasis-associated protein 2
NOTCH3: Notch receptor 3
Nrf2: Nuclear factor (erythroid-derived 2)-like 2
NBNC-HCC: Non-hepatitis B / non-C hepatocellular carcinoma
PTEN: Phosphatase and tensin homolog
p38MAPK: P38 mitogen-activated protein kinase
PIK3CA: Phosphatidylinositol‐4, 5‐bisphosphate 3‐kinase catalytic subunit alpha
R/R AML: Refractory or relapsed acute myeloid leukemia
RND3: Rho family GTPase 3
ROCK: Rho associated coiled‐coil containing protein kinase
ROCK1: Rho-associated coiled-coilcontaining protein kinase 1
SOX4: Sex determining region Y-box 4
STAT3: Signal transducer and activator of transcription 3
SMC1A: Structural maintenance of chromosomes protein 1A
SHNG3: Small nucleolar RNA host gene 3
SNHG5: Small nucleolar RNA host gene 5
TUG1: Taurine up-regulated gene 1
VEGF: Vascular endothelial growth factor
YAP: Yes‐associated protein
ZEB2: Zinc finger E-box binding homeobox 2
2-DG: 2-Deoxy-d-glucose
